# Effects of Pen Partition Design and Hiding Facilities on Elimination and Lying Behavior of Finishing Pigs

**DOI:** 10.3390/ani16050788

**Published:** 2026-03-03

**Authors:** Zhou Yu, Hao Wang, Zhi He, Bin Hu, Renli Qi, Yaqiong Zeng

**Affiliations:** 1 Chongqing Academy of Animal Sciences, Chongqing 402460, China; 2 National Center of Technology Innovation for Pigs, Chongqing 402460, China

**Keywords:** elimination behavior, finishing pigs, hiding facility, lying behavior, partition type

## Abstract

In intensive commercial pig production systems, the spatial distribution of elimination and lying behaviors plays a crucial role in pen hygiene, management efficiency, and animal welfare. Pen partition design and the provision of hiding facilities are key structural factors that may influence pigs’ spatial preferences; however, systematic evaluations of their combined effects remain limited. A total of 108 growing–finishing pigs were used in a 3 × 2 factorial design to assess the effects of different partition types and hiding facility configurations, as well as their interaction, on the spatial distribution of elimination and lying behaviors. Behavioral data were analyzed using non-parametric statistical methods. The results showed that partition type and hiding facilities significantly influenced the spatial patterns of elimination and lying behaviors (*p* < 0.05), whereas no significant effects were observed on total daily elimination duration, elimination frequency, or lying posture distribution (*p* > 0.05). Elimination behavior was predominantly concentrated in the slatted floor area. The combination of a front-closed partition with a hiding facility significantly increased the proportion of disturbed elimination events (16.2 ± 14.3%), which was higher than that observed in the open partition combined with a hiding facility (7.9 ± 7.6%, *p* < 0.05). In contrast, the rear-closed partition design was associated with atypical elimination occurring on the solid floor area. Overall, pigs showed a clear preference for lying on the solid floor. The front-closed partition combined with a hiding facility significantly reduced the proportion of lying on the solid floor (64.6 ± 8.5%), whereas the open partition combined with a hiding facility resulted in a higher-than-average proportion of solid-floor lying behavior (80.6 ± 8.9%). These findings indicate that an open partition design combined with a hiding facility is more effective in maintaining functional separation between elimination and resting areas while reducing disturbed elimination events. This study provides experimental evidence to support structural optimization of growing–finishing pig housing, contributing to improved pen hygiene and enhanced animal welfare.

## Simple Summary

The spatial distribution of elimination and lying areas within pig pens directly affects pen cleanliness, management efficiency, and animal welfare. Different partition designs and the provision of hiding facilities may alter how pigs allocate space for resting and elimination. In this study, growing–finishing pigs were systematically observed to evaluate the effects of various partition types and hiding facility combinations on the spatial distribution of elimination and lying behaviors. The results indicate that an appropriate combination of partition structure and hiding facilities helps maintain a clear functional separation between elimination and resting areas, thereby reducing disturbed elimination events and spatial disorder. Notably, the open partition combined with a hiding facility showed more stable spatial utilization patterns, whereas the front-closed partition combined with a hiding facility increased the risk of overlap between elimination and lying areas. These findings provide behavioral evidence to support structural optimization in commercial growing–finishing pig housing. Proper pen design can reduce pen contamination, stabilize functional zoning, improve hygiene conditions, and ultimately enhance animal welfare.

## 1. Introduction

In intensive commercial pig production systems, the spatial distribution of elimination and lying behaviors within pens is a fundamental behavioral factor affecting pen hygiene, management efficiency, and animal welfare [1,2]. Pigs naturally exhibit a tendency to separate elimination and resting areas [3], typically choosing to lie and rest away from soiled locations. This behavioral characteristic helps maintain cleaner resting areas, thereby reducing fecal contamination and pathogen transmission risks [4,5,6]. Previous studies have shown that when functional zones within a pen are clearly defined and environmental conditions are appropriate, pigs can establish relatively stable spatial patterns of elimination and lying behavior.

The spatial choice of elimination and lying behavior is highly dependent on the physical characteristics of the housing environment, including floor type, temperature gradients, ventilation conditions, lighting, and spatial visibility [7,8]. Pigs generally prefer to eliminate in relatively cooler and well-ventilated areas, while selecting dry, semi-enclosed, and less disturbed areas for lying [9,10]. When marked differences in temperature or airflow exist within a pen, pigs may adjust the locations of elimination and resting areas accordingly. Therefore, housing design plays a critical role in shaping and stabilizing behavioral functional zones.

Partition structure is one of the key engineering factors influencing the spatial attributes of a pen. Different degrees of partition closure can alter airflow patterns, light distribution, and visual permeability, and may also affect social interaction frequency and movement pathways, thereby indirectly influencing the spatial allocation of elimination and lying behaviors [11]. Some studies suggest that partially enclosed partitions may improve pen cleanliness under certain conditions, but restricted ventilation or blurred spatial boundaries may also induce shifts in elimination areas [12]. However, current findings regarding the effects of different partition closure types on the spatial organization of elimination and lying behaviors in growing–finishing pigs remain inconsistent.

In addition to partition design, hiding facilities are commonly installed within pens to alleviate social stress and reduce aggressive or abnormal behaviors [13,14]. By providing visual barriers and temporary refuge spaces, hiding facilities may alter pigs’ spatial occupancy patterns and distribution of time spent in specific areas, potentially influencing the locations of elimination and lying behaviors [15]. Nevertheless, most existing studies have focused on the effects of hiding facilities on social or aggressive behaviors, while limited attention has been paid to their potential influence on the spatial distribution of elimination and lying behaviors.

Importantly, partition closure types and hiding facilities often coexist in practical production settings; however, systematic investigations of their combined effects remain scarce. Whether different partition structures modify the stability of elimination and resting areas in the presence of hiding facilities, increase behavioral disturbances, or blur functional zoning has not yet been thoroughly examined. This knowledge gap limits the scientific application of pen structural design in commercial pig production.

## 2. Materials and Methods

### 2.1. Experimental Housing and Animals

**Figure 1:**
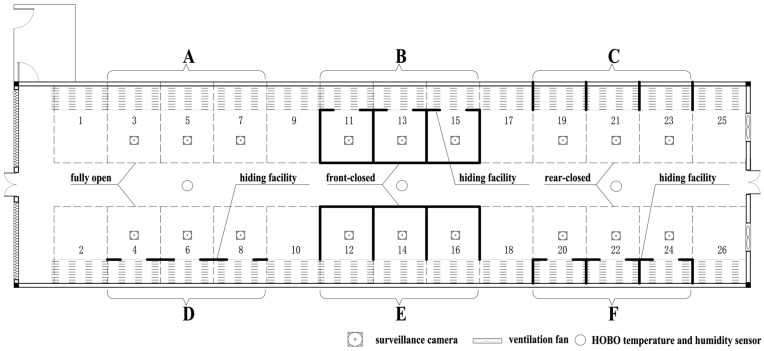
Layout of the experimental pen arrangement.

The experiment was conducted from July to August at the Livestock Behavior and Environmental Regulation Experimental Base of the Chongqing Academy of Animal Sciences, China. The study was intentionally performed during summer because, under the subtropical hilly climate of Chongqing, this period is characterized by high ambient temperature and heat load. Under elevated thermal conditions, pigs’ spatial allocation of elimination and lying behavior is more sensitive to environmental variation, allowing structural effects of pen design to be more clearly identified. The experimental building (No. 5 pig house) consisted of 26 pens arranged in two east–west rows separated by a central corridor. Slatted floors (700 mm width) were located along both the north and south sides of each pen, with a floor slope of 3%. A drinker was installed in the corner of the slatted floor area (Figure 1).

Eighteen standard open pens (3.0 m × 3.08 m) were selected for modification, including nine pens on each side of the corridor (pens 3, 5, 7, 11, 13, 15, 19, 21, 23 and pens 4, 6, 8, 12, 14, 16, 20, 22, 24). Adjacent treatment pens were separated by one empty pen to minimize cross-interference. A mechanical ventilation system was used throughout the experiment, and ventilation mode and operating parameters were kept consistent across all pens.

A total of 108 growing–finishing pigs (Duroc × Landrace × Yorkshire) were used. Before allocation, pigs were balanced according to body weight and sex and then randomly assigned to treatments to ensure similar sex ratios and age ranges among groups. All pigs originated from the same experimental herd and had comparable health status at the start of the experiment, thereby minimizing potential confounding effects related to genetic background, age, and source.

### 2.2. Experimental Design

Eighteen of the 26 pens were used in the experiment, with six pigs per pen (108 pigs in total). Pigs were fed twice daily at fixed times (08:00–09:00 and 15:00–16:00). Pen cleaning was performed once daily at 09:00 using the same procedure and frequency across all pens to reduce potential management-related variation in elimination behavior.

A 3 × 2 factorial design was employed, consisting of three partition types and two hiding facility conditions (Groups A–F), with three replicate pens per treatment.

- Group A (control): open partition + no hiding facility.
- Group B: front-closed partition + hiding facility.
- Group C: rear-closed partition + no hiding facility.
- Group D: open partition + hiding facility.
- Group E: front-closed partition + no hiding facility.
- Group F: rear-closed partition + hiding facility.

Both partition panels and hiding facilities were constructed from stainless steel solid plates welded onto the pen structure. The pen height was 1000 mm, and the solid panel height was 500 mm, with a thickness of 2 mm. Materials and installation methods were consistent within each type. The panels primarily functioned as visual barriers and partial airflow modifiers.

**Figure 2:**
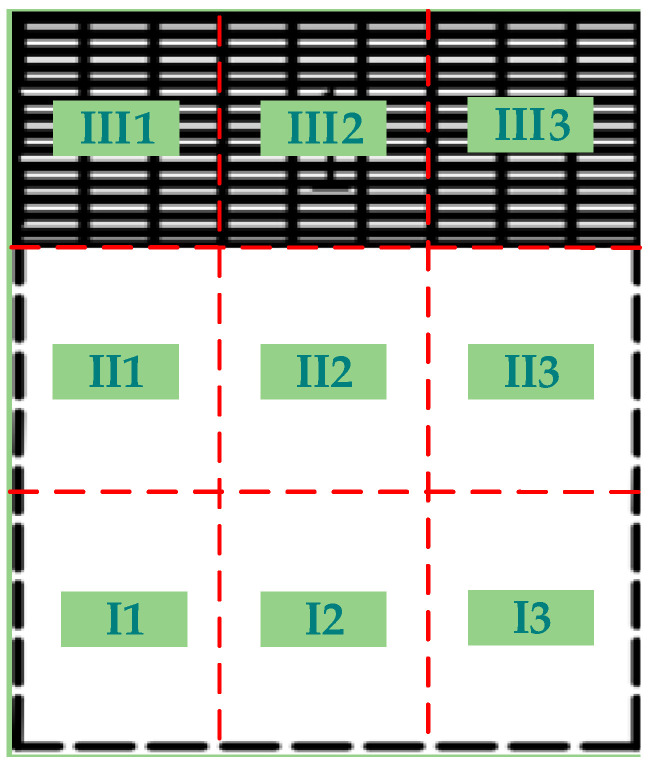
Schematic diagram of pen zone division.

Each pen was divided into nine spatial zones for behavioral analysis (Figure 2). Based on the orientation from the central corridor toward the slatted floor area, the pen was divided longitudinally into front, middle, and rear sections and laterally into left, central, and right sections, forming a 3 × 3 grid.

For elimination behavior, the location of each elimination event was recorded and summarized to calculate the proportion of elimination occurring in each zone. The elimination area was defined as the combination of zones accounting for more than 80% of total elimination events, allowing assessment of spatial location and area proportion under different pen configurations.

For lying behavior, if a pig occupied more than one zone simultaneously, the zone containing the majority of the body was recorded. If body distribution was approximately equal, the zone containing the head was used for classification.

- The slatted-floor area (Zone III) refers to the combined zones III1, III2, and III3, while the slatted-floor corner area refers specifically to zones III1 and III3.
- The solid-floor area (Zones I + II) includes zones I1, I2, I3, II1, II2, and II3.
- The solid-floor corner area refers to zones I1 and I3, and the central intermediate area refers to zones II1 and II3.

### 2.3. Measurements and Behavioral Recording

1. Environmental Monitoring

During the experimental period, environmental parameters including ambient temperature, relative humidity, and dew point were recorded inside the pig house. Three sensor locations were installed to comprehensively capture the thermal environment within the pigs’ activity space: near the entrance (cooling pad end), near the driving alley (fan end), and in the middle of the building.

Temperature and humidity were measured using HOBO MX230 data loggers (Onset Computer Corporation, Bourne, MA, USA). Sensors were installed at a height of 1.1 m above the floor to avoid direct contact and damage by pigs. Environmental data were recorded at 15 min intervals throughout the experiment.

2. Measurement of Elimination and Lying Behaviors

Elimination behavior was recorded through continuous 24 h video playback and manual observation. To minimize observer bias, all behavioral recordings (elimination and lying behaviors) were conducted by the same trained researcher following standardized procedures.

A high-definition camera (DS-2CD3146FWD-I, Hikvision Digital Technology Co., Ltd., Hangzhou, China) was installed above each experimental pen, ensuring full visual coverage of the pen area. Continuous video recording was performed from 00:00 to 23:59 every day for three consecutive days in the middle stage of the experiment as the core observation period. Each pen housed six pigs. Observed variables included elimination behavior and its related parameters, spatial location of elimination, disturbed elimination behavior, as well as the frequency and duration of abnormal behaviors; meanwhile, the lying location and lying posture of pigs were also recorded.

Due to limitations in camera angle and image resolution, urination and defecation were not distinguished and were collectively classified as elimination behavior.

Lying behavior was recorded using the instantaneous scan sampling method [16], which is suitable for long-duration state behaviors and can well reflect the structural characteristics of behavioral time allocation. According to the circadian activity rhythm of pigs, differentiated observation frequencies were set:

- During the active period (07:00–20:00), observations were recorded every 10 min;
- During the resting period (00:00–07:00 and 20:00–24:00), observations were recorded every 30 min.

Because lying is a long-duration behavior, only the proportion of lying frequency and lying posture were analyzed, while total lying duration was not calculated. Lying postures [17] were categorized into three types:

- Sternal recumbency: most limbs tucked beneath the body, dorsal surface facing upward;
- Lateral recumbency: one side of the body in contact with the floor, limbs visible;
- Semi-Sternal recumbency: body partially supported laterally with partial limb extension.

Disturbed elimination was defined as any social contact from pen mates occurring during the elimination process, including nose contact, biting, pushing, or body collisions. Only disturbances occurring between the onset and termination of an elimination event were recorded.

### 2.4. Data Processing and Statistical Analysis

Behavioral data collected by cameras over three consecutive days in the middle stage of the experiment were statistically compiled by researchers, who recorded the elimination and lying behaviors of finishing pigs. Microsoft Excel was used to collate and summarize the original data including the onset and termination time, frequency, spatial location of elimination and lying behaviors, as well as lying postures. In this study, the pen was used as the experimental unit for data processing and statistical analysis. First, the overall mean value of each behavioral indicator for all pigs in a single pen was calculated over the three days to complete the aggregation of raw data at the pen level, and the independent aggregated mean value of each behavioral indicator corresponding to the treatment group of each pen was obtained.

Statistical analysis was conducted on the aggregated mean data at the pen level using Python (version 3.10) scripts with the SciPy (version 3.11) and Statsmodels (version 0.14) libraries. Results are presented as mean ± standard deviation (SD). Since the data in this study were aggregated with the pen as the independent experimental unit and a large number of behavioral indicators were proportional data, some indicators did not meet the assumptions of normality and homogeneity of variance for parametric tests after verification. Meanwhile, the sample size of pens in each treatment group was evenly distributed, the observed values between pens were independent of each other, and the core focus of this study was to compare the relative differences in behavioral indicators among different treatment groups rather than estimate absolute parameters. Non-parametric statistical methods can achieve robust testing of inter-group differences without relying on the assumptions of data distribution, which fully meets the requirements of statistical analysis in this study.

Therefore, non-parametric statistical methods were applied for data analysis, specifically:

1. The Kruskal–Wallis H test was used to compare the aggregated mean indicators of elimination and lying behaviors at the pen level among different pen groups to analyze the inter-group differences;
2. The Scheirer–Ray–Hare test [18,19] was applied to evaluate the effects of partition type, hiding facility and their interaction on the aggregated mean indicators of elimination and lying behaviors at the pen level. Statistical significance was set at *p* < 0.05.

## 3. Results

### 3.1. Effects of Pen Group on Elimination Duration, Frequency and Disturbance

There were no significant differences among pen groups in total daily elimination duration or total elimination frequency (*p* > 0.05). On average, finishing pigs exhibited 8.5 ± 4.7 elimination events per pig per day, with a mean total elimination duration of 264.5 ± 144.8 s per pig per day (Table 1). However, significant differences were observed among groups in the proportion of disturbed elimination events (*p* < 0.05). Group B showed the highest proportion of disturbed elimination frequency (16.2 ± 14.3%), which was significantly higher than that in Group D (7.9 ± 7.6%), while the remaining groups presented intermediate values without significant differences. Similarly, the proportion of disturbed elimination duration differed significantly among groups *(p* < 0.05). Group B (9.8 ± 10.3%) was significantly higher than Group D (3.8 ± 4.8%).

### 3.2. Effects of Pen Group on the Spatial Distribution of Elimination Behavior

**Figure 3:**
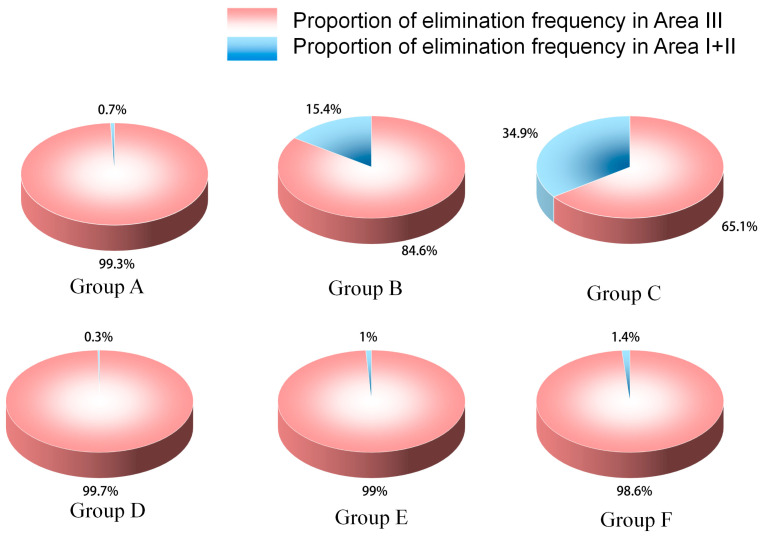
Spatial proportion of excretion frequency in finishing pigs under different pen grouping conditions.

Spatial variation in elimination behavior reflects the stability of functional zoning and potential hygiene risks within the pen. As shown in Figure 3 and Table 2 and Table 3, elimination behavior in all groups was primarily concentrated in the slatted floor area (Zone III).

The proportion of elimination duration and frequency in Zone III and Zones III1 + III3 in Group C was significantly lower than in Group D (*p* < 0.05). In contrast, the proportion of elimination duration and frequency in Zones II1 + II3 was significantly higher in Group C compared with other groups (*p* < 0.05), indicating that the rear-closed partition design may increase elimination events in the central solid-floor area.

No significant differences were observed among groups for elimination in Zones I1 + I3 (*p* > 0.05). However, descriptively, Group B showed markedly higher values than other groups, while elimination in this area was nearly absent in the remaining treatments.

### 3.3. Two-Factor Effects of Partition Type and Hiding Facility on Elimination Behavior

As shown in Table 4, partition type had a significant main effect on average single elimination duration. No significant main or interaction effects were detected for total elimination duration, elimination frequency, disturbed elimination indices, or spatial distribution indicators (*p* > 0.05).

These results indicate that different structural combinations had limited influence on overall elimination levels but may regulate elimination behavior at the level of individual event duration.

### 3.4. Effects of Pen Group on Spatial Distribution of Lying Frequency and Lying Posture

**Figure 4:**
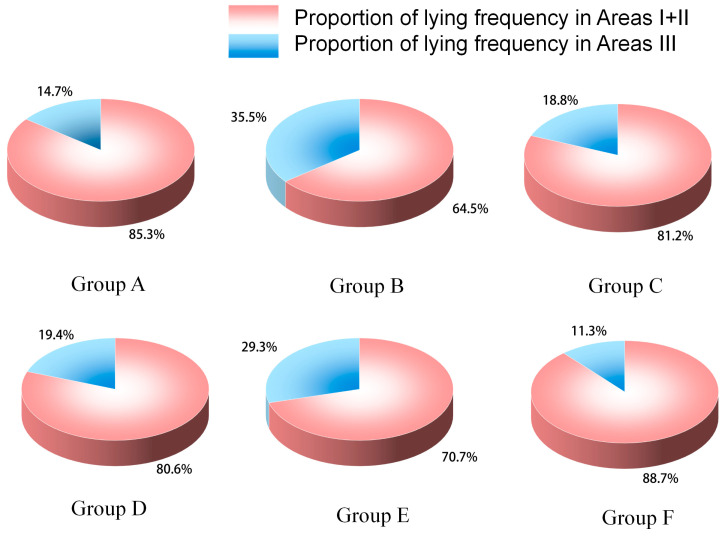
Spatial proportion of lying frequency in finishing pigs under different pen grouping conditions.

As shown in Figure 4 and Table 5, pigs in all groups predominantly lay in Zones I + II (solid-floor areas), with an overall average proportion of 76.6 ± 13.5%.

Group F showed the highest proportion of lying in Zones I + II (88.7 ± 15.7%), which was significantly higher than that of Group B (64.6 ± 8.5%, *p* < 0.05). This suggests that the combination of a front-closed partition and hiding facility reduced pigs’ preference for lying in solid-floor areas.

For Zone I1 + I3, Group C was significantly lower than Group B. For Zone II1 + II3, Group C was significantly lower than Group F. For Zone III1 + III3, Group B was significantly higher than Groups A and F.

Regarding lying posture, no significant differences were found among groups in the proportion of sternal, semi-sternal, or lateral recumbency (*p* > 0.05). Lateral recumbency remained the predominant posture across treatments, accounting for 70.9 ± 9.5% on average.

### 3.5. Two-Factor Effects of Partition Type and Hiding Facility on Lying Behavior

As shown in Table 6, partition type had significant main effects on the proportion of lying frequency in Zones I1 + I3 and II1 + II3 (*p* < 0.001).

Hiding facilities showed significant main effects on lying frequency in Zones I + II (*p* = 0.010), I1 + I3 (*p* = 0.004), and III1 + III3 (*p* = 0.009).

No significant interaction effects between partition type and hiding facility were detected for any lying spatial or posture indicators (*p* > 0.05).

These findings indicate that partition design and hiding facilities mainly influenced spatial selection of lying areas through independent main effects, while their interaction and effects on posture type were limited.

## 4. Discussion

### 4.1. Effects of Pen Configuration on Elimination Duration, Frequency, and Spatial Distribution

The present study demonstrated that different pen configurations did not significantly affect total elimination duration, total elimination frequency, or average single elimination duration in growing–finishing pigs. This suggests that elimination, as a physiologically driven behavior, maintains a relatively stable occurrence intensity across structural conditions. In contrast, its spatial distribution appears more susceptible to environmental modulation.

When examining Table 1, Table 2 and Table 3, Group C exhibited the highest total elimination duration and frequency among treatments; however, the proportion of elimination occurring in Zone III (slatted floor area) was significantly lower than that in the fully open Group A, while elimination in Zones II1 + II3 (central solid-floor area) was significantly higher than in other groups. This pattern—stable total elimination but spatial relocation—indicates that the rear-closed partition design may have weakened the functional dominance of the slatted floor as the primary elimination area, leading to partial displacement of elimination toward adjacent solid-floor zones.

One plausible explanation is that the rear-closed structure may have locally restricted airflow around the slatted floor, resulting in heat and moisture accumulation. Aarnink et al. [20] reported that when airflow is obstructed and warm, humid conditions accumulate in specific pen areas, pigs tend to avoid those locations and shift elimination toward areas with more favorable environmental conditions. Similarly, Bonn et al. [21] observed that pigs prefer to eliminate in well-ventilated areas while avoiding zones with excessive air velocity during resting.

Although environmental control systems were used in the present study to maintain temperature differences within 2 °C between front and rear sections of the building, the overall mean temperature remained relatively high (26.53 °C), with an average humidity of 78.59% during the summer trial. However, local microclimatic parameters (e.g., airflow velocity, ammonia or hydrogen sulfide concentration) were not directly measured at the pen level. Therefore, the interpretation that the rear-closed structure impaired ventilation and reduced the attractiveness of the slatted floor as a traditional elimination site remains inferential rather than directly evidenced.

Elmore et al. [22,23] demonstrated that growing–finishing pigs are capable of functionally separating resting and elimination areas when environmental conditions permit, reflecting inherent stability in elimination site preference. Compared with Group C, Group A showed lower total elimination frequency and duration, with a higher concentration of elimination in the slatted floor area. The more open partition design likely provided improved airflow uniformity and greater visual permeability, reinforcing the spatial boundary of the elimination zone and reducing the likelihood of spatial drift. Li et al. [24,25] similarly suggested that while partition type may not directly determine elimination location, greater spatial openness and clearer functional delineation contribute to maintaining elimination zone stability.

In Group B, the combination of a front-closed partition and hiding facility resulted in a markedly higher proportion of elimination in the solid corner area (Zones I1 + I3), whereas elimination in this area was nearly absent in other treatments. Although increased brightness contrast between slatted and solid areas might theoretically influence elimination choice, Randall et al. [26] reported that pigs typically rest in darker, drier areas and eliminate in brighter, wetter areas, suggesting that lighting alone cannot explain this shift.

Previous studies indicate that pigs tend to eliminate near walls or partitions, particularly in corners, possibly due to a perceived sense of protection during vulnerable elimination postures [27,28,29]. The front-closed partition combined with hiding facilities may have altered visual boundaries and spatial clarity within the pen, increasing local social interactions such as approaching, sniffing, or competition in corner areas. Consistent with this interpretation, Group B exhibited the highest proportion of disturbed elimination events and disturbed elimination duration. Increased social interference during elimination may fragment elimination bouts and elevate the likelihood of elimination occurring in atypical zones adjacent to resting areas.

Collectively, these findings suggest that while elimination intensity remains physiologically regulated, pen structure can influence the spatial organization and stability of elimination zones. Structural designs that obscure spatial boundaries or increase social disturbance may elevate the risk of functional overlap between elimination and resting areas, thereby potentially compromising hygiene stability.

### 4.2. Structural Influence of Partition Type and Hiding Facility on Elimination Patterns

The two-factor analysis revealed that, except for average single elimination duration, neither partition type, hiding facility, nor their interaction significantly affected total elimination intensity or spatial proportion indicators (*p* > 0.05). This indicates that under the present experimental conditions, pen structure and hiding facility combinations did not systematically alter the overall occurrence of elimination behavior.

From a biological perspective, elimination frequency is strongly driven by feeding rhythm, metabolic status, and physiological needs. Therefore, relative stability across structural treatments is biologically plausible. Structural factors appear to exert greater influence on the spatial organization of elimination rather than on its intensity.

The significant main effect of partition type on average single elimination duration suggests that structural configuration may influence the continuity of elimination events. When spatial boundaries are unclear or social disturbance increases, elimination behavior may become more fragmented, affecting single-bout duration. However, as local microclimate parameters and direct social interaction frequencies were not quantitatively measured, interpretations regarding ventilation constraints, odor accumulation, or social disturbance mechanisms remain behavior-based inferences. Future studies integrating detailed environmental monitoring and social interaction tracking would be necessary to confirm these mechanisms.

Methodologically, separating “behavioral intensity” from “spatial distribution” in the analysis allowed clearer identification of structural effects at different regulatory levels. In practical production systems, altering elimination frequency may not be the primary objective. Instead, maintaining functional separation between elimination and resting areas and ensuring spatial stability are more critical for pen hygiene and management efficiency. Stable functional zoning can reduce fecal contact, minimize social disturbance, and contribute to improved welfare outcomes. Therefore, structural optimization should prioritize spatial organization and boundary clarity rather than focusing solely on changes in behavioral frequency.

### 4.3. Effects of Pen Configuration on the Spatial Distribution of Lying Frequency and Lying Posture

The present results indicate that although the proportion of different lying postures did not differ significantly among pen groups, the spatial distribution of lying behavior varied markedly. This suggests that pen structure primarily regulates spatial selection of lying areas rather than altering the occurrence type of lying behavior.

Overall, pigs predominantly rested on the solid-floor areas across all treatments. However, differences in the utilization of solid-floor zones were observed among groups. In Group B, the proportion of lying in Zones I + II was significantly lower than in Groups A and F, while lying in Zones III1 + III3 (adjacent to the slatted floor) was relatively higher. This pattern indicates that under the combination of a front-closed partition and hiding facility, the stability of solid corner areas as primary resting zones was reduced.

Under typical conditions, Zones I1 + I3 (solid corner areas) provide characteristics preferred by growing–finishing pigs for resting, including distance from excreta odor [30,31], enclosure, and reduced light exposure. However, the lying proportion in these zones was significantly lower in Group B compared with other groups. Combined with the elimination results (Table 3), which showed some elimination events occurring in I1 + I3 under this treatment, the functional boundary between elimination and resting areas may have become blurred. Residual odor or contamination could reduce the attractiveness of these areas for lying.

Additionally, the combination of a front-closed partition and hiding facility may have altered the spatial attributes of corner zones, increasing their use for social approaches, sniffing, or temporary occupancy. This may elevate social disturbance during resting. Supporting this interpretation, Group B showed the highest proportions of disturbed elimination frequency and duration (Table 1), suggesting greater overall social interference under this structural condition. Ekkel et al. [32] reported that pigs select lying locations not only based on floor type but also considering social disturbance and perceived safety. When disturbance intensity increases in corner areas, their stability as resting zones may decline. These interacting factors likely contributed to the redistribution of lying behavior observed in Group B.

Regarding posture, no significant differences were detected among treatments for sternal, semi-sternal, or lateral recumbency. Lateral recumbency remained the predominant posture across all groups. This indicates that structural variation had limited influence on posture selection, which is more strongly regulated by thermoregulation, circadian rhythm, and growth stage [33]. Hillmann et al. [34] reported that pigs may shift toward slatted floors when lateral recumbency is insufficient for heat dissipation, particularly under heat stress conditions [35,36]. Therefore, posture expression appears to reflect physiological state rather than partition configuration.

### 4.4. Structural Regulation of Lying Behavior Under Different Partition Types and Hiding Facilities

Two-factor analysis (Table 6) showed significant main effects of partition type on lying frequency in Zones I + II, I1 + I3, and III1 + III3, while hiding facilities exerted significant main effects on Zones I1 + I3 and II1 + II3. No significant interaction effects were detected.

The influence of partition type on the distribution of lying between solid and slatted areas suggests that structural permeability affects the clarity of resting-zone boundaries. Under more open structural conditions, pigs tended to maintain solid-floor areas as their primary resting zones. In contrast, relatively enclosed configurations appeared to induce partial redistribution of lying locations. This finding is consistent with the spatial reorganization observed in elimination behavior, further indicating that pen structure systematically influences functional zone organization.

The significant main effects of hiding facilities on Zones I1 + I3 and II1 + II3 imply that the presence of visual barriers may enhance the spatial attractiveness of certain solid-floor areas, allowing intermediate zones to partially assume resting functions. This modification of spatial use may be associated with altered visual shielding, perceived safety, or group distribution dynamics. However, as the mechanisms underlying individual spatial preference were not directly measured, these interpretations remain inferential and warrant further investigation.

Notably, posture-related indicators did not show significant main or interaction effects, reinforcing the conclusion that structural factors predominantly regulate spatial allocation rather than physiological posture expression.

Taken together, elimination and lying results reveal a consistent regulatory pattern: pen structure and hiding facilities primarily modulate the spatial organization of functional zones, while exerting limited influence on behavioral intensity or posture type. This “spatial-organization-priority” pattern carries important welfare implications. Stable functional zoning helps maintain clear boundaries between elimination and resting areas, reduces contamination risk in lying zones, and may lower fecal contact, skin soiling, and potential pathogen exposure. Clear spatial boundaries can also reduce social interference frequency, enhancing behavioral stability and perceived safety during rest.

Although this study did not directly measure cleanliness indices, health parameters, or physiological stress indicators, from a behavioral ecological perspective, functional zone stability constitutes an essential component of housing quality. Therefore, structural optimization should prioritize spatial-use patterns and boundary stability to support welfare improvement and hygiene management objectives.

## 5. Conclusions

This study systematically evaluated the combined effects of partition type and hiding facilities on elimination and lying behaviors in growing–finishing pigs. The results demonstrate that pen structure did not significantly alter the overall intensity of elimination or lying behaviors, nor lying posture distribution, but significantly influenced their spatial organization.

Specifically, the combination of a front-closed partition with a hiding facility (Group B) was associated with weakened spatial separation between elimination and resting areas, increased functional overlap in corner zones, and elevated levels of disturbed elimination, indicating reduced functional zoning stability.

In contrast, the open partition combined with a hiding facility (Group D) showed a more balanced pattern, maintaining elimination concentration in slatted areas while preserving stable resting behavior in solid-floor zones, thereby supporting clearer spatial separation between elimination and resting areas.

From a practical production perspective, functional zoning stability is directly linked to contamination risk in resting areas, cleaning efficiency, and environmental hygiene. Therefore, in commercial growing–finishing pig housing design, an open partition combined with appropriately configured hiding facilities is recommended, whereas the use of front-closed partitions in combination with additional visual barriers should be approached with caution.

This study was conducted during a single season and at one experimental site, and microclimatic parameters and health indicators were not simultaneously monitored. The underlying mechanisms therefore require further verification. Future research integrating multi-seasonal trials, individual positioning technologies, environmental monitoring, and welfare assessment indicators would help clarify the causal pathways linking structural factors, spatial organization, and long-term production and welfare outcomes.

## Figures and Tables

**Table 1:** Effects of different pen groups on elimination duration and frequency of finishing pigs.

Group	Total Elimination Duration (s/Pig/Day)	Total Elimination Frequency (Events/Pig/Day)	Mean Duration Per Elimination Event (s/Event)	Proportion of Disturbed Elimination Frequency (%)	Proportion of Disturbed Elimination Duration (%)
A	266.9 ± 66.8 a	7.9 ± 2.1 a	34.9 ± 1.6 a	11.5 ± 7.2 ab	5.2 ± 3.3 ab
B	295.3 ± 143.6 a	8.2 ± 4.4 a	37.4 ± 7.9 a	16.2 ± 14.3 a	9.8 ± 10.3 a
C	312.3 ± 126.8 a	10.8 ± 4.6 a	29.6 ± 4.3 a	15.4 ± 9.3 ab	5.2 ± 3.5 ab
D	276.2 ± 124.1 a	8.5 ± 3.8 a	32.6 ± 4.8 a	7.9 ± 7.6 b	3.8 ± 4.8 b
E	308.3 ± 146.9 a	9.9 ± 4.6 a	31.7 ± 6.1 a	8.4 ± 3.2 ab	3.4 ± 1.7 ab
F	271.1 ± 124.2 a	9.9 ± 4.1 a	27.1 ± 4.7 a	9.8 ± 4.9 ab	4.8 ± 3.9 ab

Different lowercase letters within the same column indicate significant differences among groups (*p* < 0.05).

**Table 2:** Effects of different pen groups on the spatial distribution of elimination duration.

Group	Proportion of Elimination Duration in Area III (%)	Proportion of Elimination Duration in Areas III1 + III3 (%)	Proportion of Elimination Duration in Areas II1 + II3 (%)	Proportion of Elimination Duration in Areas I1 + I3 (%)
A	99.8 ± 3.6 a	94.1 ± 9.9 ab	0.1 ± 0.3 b	0 a
B	84.3 ± 24.9 a	81.4 ± 26.4 ab	6.6 ± 9.9 b	9.1 ± 16.1 a
C	61.2 ± 31.9 b	63.2 ± 29.2 b	35.2 ± 29.8 a	0 a
D	99.7 ± 0.8 a	98.9 ± 2.1 a	0.3 ± 0.6 b	0 a
E	99.0 ± 2.0 a	96.0 ± 3.6 a	1.3 ± 2.5 b	0 a
F	98.6 ± 2.6 a	95.2 ± 4.4 a	1.6 ± 2.6 b	0.3 ± 0.8 a

Note: Different lowercase letters within the same column indicate significant differences among groups (*p* < 0.05). The proportion of elimination duration in Area III was calculated as the sum of elimination duration in Zones III1, III2, and III3 divided by the total elimination duration across Areas I, II, and III. The proportion of elimination duration in Areas III1 + III3 was calculated as the sum of elimination duration in Zones III1 and III3 divided by the total elimination duration across Areas I, II, and III. The proportion of elimination duration in Areas I1 + I3 and II1 + II3 was calculated using the same method. The calculation method for lying spatial indicators was analogous.

**Table 3:** Effects of different pen groups on the spatial distribution of elimination frequency.

Group	Proportion of Elimination Frequency in Area III (%)	Proportion of Elimination Frequency in Areas III1 + III3 (%)	Proportion of Elimination Frequency in Areas II1 + II3 (%)	Proportion of Elimination Frequency in Areas I1 + I3 (%)
A	99.3 ± 1.5 a	91.1 ± 13.4 a	0.6 ± 1.4 b	0 a
B	84.6 ± 25.1 ab	82.39 ± 25.43 ab	5.5 ± 8.4 b	9.2 ± 15.9 a
C	65.1 ± 29.6 b	67.4 ± 25.9 b	29.7 ± 27.6 a	0 a
D	99.6 ± 1.3 a	98.2 ± 3.6 a	0.4 ± 0.8 b	0 a
E	99.1 ± 1.8 a	94.9 ± 4.1 a	1.1 ± 1.9 b	0 a
F	98.5 ± 1.9 ab	94.4 ± 5.1 ab	1.4 ± 1.7 b	0.4 ± 0.8 a

Different lowercase letters within the same column indicate significant differences among groups (*p* < 0.05).

**Table 4:** Two-factor analysis: Partition Types × Hiding Facility on elimination behavior of finishing pigs.

Elimination Index	Partition Type		Hiding Facility		Partition Type × Hiding Facility	
	*p* Value	F Value	*p* Value	F Value	*p* Value	F Value
Total elimination duration (s/pig/day)	0.607	0.001	0.372	0.066	0.645	0.131
Total elimination frequency (events/pig/day)	0.621	0.952	0.938	0.006	0.841	0.346
Proportion of disturbed elimination frequency (%)	0.804	0.436	0.627	0.236	0.526	1.284
Proportion of disturbed elimination duration (%)	0.930	0.146	0.413	0.671	0.454	1.578
Proportion of elimination frequency in Area III (%)	0.358	2.056	0.810	0.058	0.459	1.515
Proportion of elimination duration in Area III (%)	0.320	2.276	0.828	0.047	0.476	1.484
Mean duration per elimination frequency (s/event)	0.029 *	7.11	0.370	0.805	0.122	4.208

Note: * *p* < 0.05. F denotes the F-statistic of the analysis of variance.

**Table 5:** Effects of different pen groups on the spatial distribution and types of lying behavior of finishing pigs.

Group	Proportion of Lying Frequency in Areas I + II (%)	Proportion of Lying Frequency in Areas I1 + I3 (%)	Proportion of Lying Frequency in Areas II1 + II3 (%)	Proportion of Lying Frequency in Areas III1 + III3 (%)	Proportion of Sternal Recumbency Frequency (%)	Proportion of Semi-Sternal Recumbency Frequency (%)	Proportion of Lateral Recumbency Frequency (%)
A	85.3 ± 12.1 a	42.8 ± 13.9 ab	27.5 ± 6.6 ab	11.8 ± 10.8 b	17.9 ± 7.8 a	10.2 ± 11.1 a	72.2 ± 13.3 a
B	64.6 ± 8.5 b	27.1 ± 9.1 b	29.6 ± 9.9 ab	29.8 ± 7.0 a	16.7 ± 6.1 a	9.0 ± 8.4 a	72.7 ± 11.9 a
C	81.2 ± 7.6 ab	47.6 ± 4.9 a	18.6 ± 3.7 b	14.9 ± 6.2 ab	19.6 ± 5.1 a	8.9 ± 6.8 a	71.4 ± 7.5 a
D	80.6 ± 8.9 ab	35.1 ± 5.8 ab	31.4 ± 6.9 a	16.7 ± 7.9 ab	23.8 ± 8.1 a	8.5 ± 6.9 a	67.8 ± 6.8 a
E	70.7 ± 13.9 ab	37.3 ± 7.1 ab	24.3 ± 5.1 ab	21.9 ± 11.2 ab	24.5 ± 10.0 a	5.3 ± 3.3 a	70.1 ± 9.5 a
F	88.7 ± 15.7 a	41.2 ± 6.2 a	29.2 ± 6.6 a	12.9 ± 14.1 b	20.5 ± 7.4 a	7.2 ± 4.9 a	71.7 ± 10.6 a

Different lowercase letters within the same column indicate significant differences among groups (*p* < 0.05).

**Table 6:** Two-factor analysis: Partition Types × Shelter Facilities on lying behavior of finishing pigs.

Lying Index	Partition Type		Hiding Facility		Partition Type × Hiding Facility	
	*p* Value	F Value	*p* Value	F Value	*p* Value	F Value
Proportion of lying frequency in Areas I + II (%)	0.010 *	9.216	0.200	1.640	0.382	1.924
Proportion of lying frequency in Areas I1 + I3 (%)	0.004 **	10.875	<0.001 **	13.266	0.946	0.112
Proportion of lying frequency in Areas II1 + II3 (%)	0.580	2.548	<0.001 **	13.103	0.319	2.287
Proportion of lying frequency in Areas III1 + III3 (%)	0.009 **	9.364	0.248	1.332	0.446	1.617
Proportion of sternal recumbency frequency (%)	0.942	0.119	0.944	0.005	0.283	2.522
Proportion of semi-sternal recumbency frequency (%)	0.903	0.816	0.832	0.045	0.733	0.621
Proportion of lateral recumbency frequency (%)	0.935	0.134	0.723	0.126	0.729	0.632

Note: * *p* < 0.05; ** *p* < 0.01.

## Data Availability

The original contributions presented in this study are included in the article. Further inquiries can be directed to the corresponding authors.
